# Recent Trends in Maternal and Postpartum Suicide and Countermeasures

**DOI:** 10.31662/jmaj.2021-0221

**Published:** 2022-03-11

**Authors:** Jun Takeda, Satoru Takeda, Wakako Hikiji

**Affiliations:** 1 Department of Obstetrics and Gynecology, Juntendo University Faculty of Medicine, Tokyo, Japan; 2 Aiiku Research Institute for Maternal, Child Health and Welfare, Imperial Gift Foundation Boshi-Aiiku-Kai, Tokyo, Japan; 3 Tokyo Medical Examiner's Office, Tokyo Metropolitan Government, Tokyo, Japan

**Keywords:** COVID-19, Postpartum depression, Pregnancy, Suicide

On 2017, Takeda S. reported with the data from the Tokyo Medical Examiner’s Office that suicide was the leading cause of death among pregnant and postpartum women, including up to one year after giving birth ^(1)^. Since then, various further efforts have been made to reduce suicide, and on July 25, 2017, Japan’s Cabinet approved “Overview of Comprehensive Measures to Prevent Suicide” ^(2)^.

The graph created by the Ministry of Health, Labor and Welfare from the National Police Agency’s suicide statistics original data and the Ministry of Internal Affairs and Communications’ population estimates and the national census data shows that the number of suicides has been steadily decreasing since 2011 ^(3)^. However, looking at the data over the last two years, we can see that the suicide rate has increased slightly in some generations. A closer look at the data shows that the number of male suicides in 2020 decreased by 23 cases compared to 2019 (14078 cases in 2019, 14055 cases in 2020), whereas the number of female suicides increased by 935 cases (6091 cases in 2019, 7026 cases in 2020). Suicides among the younger generation are increasing, especially among females in their 20s and teens, with an increase of 3.3% and 1.1%, respectively. The COVID-19 pandemic over the past two years may have contributed to the increase in the number of suicides among the younger generation by creating to a sense of isolation owing to movement restrictions and vague anxiety.

According to the data from the Tokyo Medical Examiner’s Office, the number of suicides among pregnant and postpartum women during the 10-year period from 2005 to 2014 was 63, or 8.7 per 100,000 live births, compared to 25, or 5.4 per 100,000 live births from 2015 to 2020 (Table 1) ^(1), (4)^. It can be stated that the suicide rate of pregnant and postpartum women has been decreasing. In addition, the aforementioned increase in the suicide rate among young females over the last two years, which includes nonpregnant women, has not been found in pregnant and postpartum women, and the data appears to be almost flat. This is assumed to be due to the positive effects of increased attention to pregnant and postpartum women, greater understanding of the importance of postpartum care, and the fact that two-week checkups are now subsidized in some areas. We believe that the overall direction of maternal suicide prevention is not wrong, but it goes without saying that further measures are needed in Japan, where the suicide rate of pregnant and postpartum women is still higher than in other countries ^(1)^.

**Table 1.** Survey of Abnormal Maternal Deaths in 23 Wards of Tokyo (2015-2020).

Year	Number of maternal deaths*	per 100,000 live births
2015	5	6.2
2016	3	3.7
2017	3	3.8
2018	4	5.2
2019	5	6.8
2020	5	6.9
Total	25	5.4

*Maternal deaths include death during pregnancy and up to one year postpartum.

In a situation where the pandemic of COVID-19 has not been completely eliminated, it will be necessary to continue to undertake further suicide control measures, as many people are likely to feel anxious. The measures should be undertaken especially for the postpartum women, among whom the suicide rates were lower as compared to the pregnant women. In this sense, the partial amendment to the Maternal and Child Health Act that came into effect in April 2021 ^(5)^, obligating municipalities to undertake efforts to provide postpartum care services, is considered as a good move. Furthermore, in 2021, private companies launched an insurance policy that covers postpartum depression. Keeping a careful watch on postpartum care may reduce the number of postpartum suicides, and this is something that we need to continue to focus on.

## Article Information

### Conflicts of Interest

None

### Author Contributions

JT prepared the first draft. WH provided the data. ST and WH revised the manuscript. All authors have read and approved the final version of the manuscript.

### Approval by Institutional Review Board (IRB)

N/A
